# Supplementary material for: Construction and verification of a prognostic model for bladder cancer based on disulfidptosis-related angiogenesis genes
Source: PeerJ. 2025 Feb 21;13:e18911. doi: 10.7717/peerj.18911 (PMC11849515; doi:10.7717/peerj.18911)
Supplement: Supplemental Information 1 [file peerj-13-18911-s001.docx]

**Table S1: Clinical information of the TCGA cohort**

| **TCGA-BLCA cohort（N=401）** | **number** |
| --- | --- |
| **Age** |  |
| <65 | 149 |
| >=65 | 252 |
| **Gender** |  |
| male | 297 |
| female | 104 |
| **Stage** |  |
| I | 2 |
| II | 124 |
| III | 141 |
| IV | 132 |
| **T** |  |
| 1 | 3 |
| 2 | 115 |
| 3 | 193 |
| 4 | 61 |
| **N** |  |
| 0 | 236 |
| 1 | 46 |
| 2 | 76 |
| 3 | 7 |
| **M** |  |
| 0 | 194 |
| 1 | 10 |
| **Race** |  |
| White | 320 |
| Asian | 43 |
| Black or african american | 22 |
| Not reported | 16 |
