# Supplementary material for: Construction and verification of a prognostic model for bladder cancer based on disulfidptosis-related angiogenesis genes
Source: PeerJ. 2025 Feb 21;13:e18911. doi: 10.7717/peerj.18911 (PMC11849515; doi:10.7717/peerj.18911)
Supplement: Supplemental Information 2 [file peerj-13-18911-s002.docx]

**Table S2: Clinical information of the GEO cohort**

| **GSE32548 cohort (N=130)** | **number** |
| --- | --- |
| **Age** |  |
| <65 | 37 |
| >=65 | 93 |
| **Gender** |  |
| male | 99 |
| famale | 31 |
| T |  |
| Ta | 40 |
| T1 | 52 |
| T2 | 38 |
| **Grade** |  |
| G1 | 15 |
| G2 | 40 |
| G3 | 75 |
