# Supplementary figures and images for: Construction and verification of a prognostic model for bladder cancer based on disulfidptosis-related angiogenesis genes
Source: PeerJ. 2025 Feb 21;13:e18911. doi: 10.7717/peerj.18911 (PMC11849515; doi:10.7717/peerj.18911)

**Figure S1:The calibration plot of the nomogram(A-C)**

**
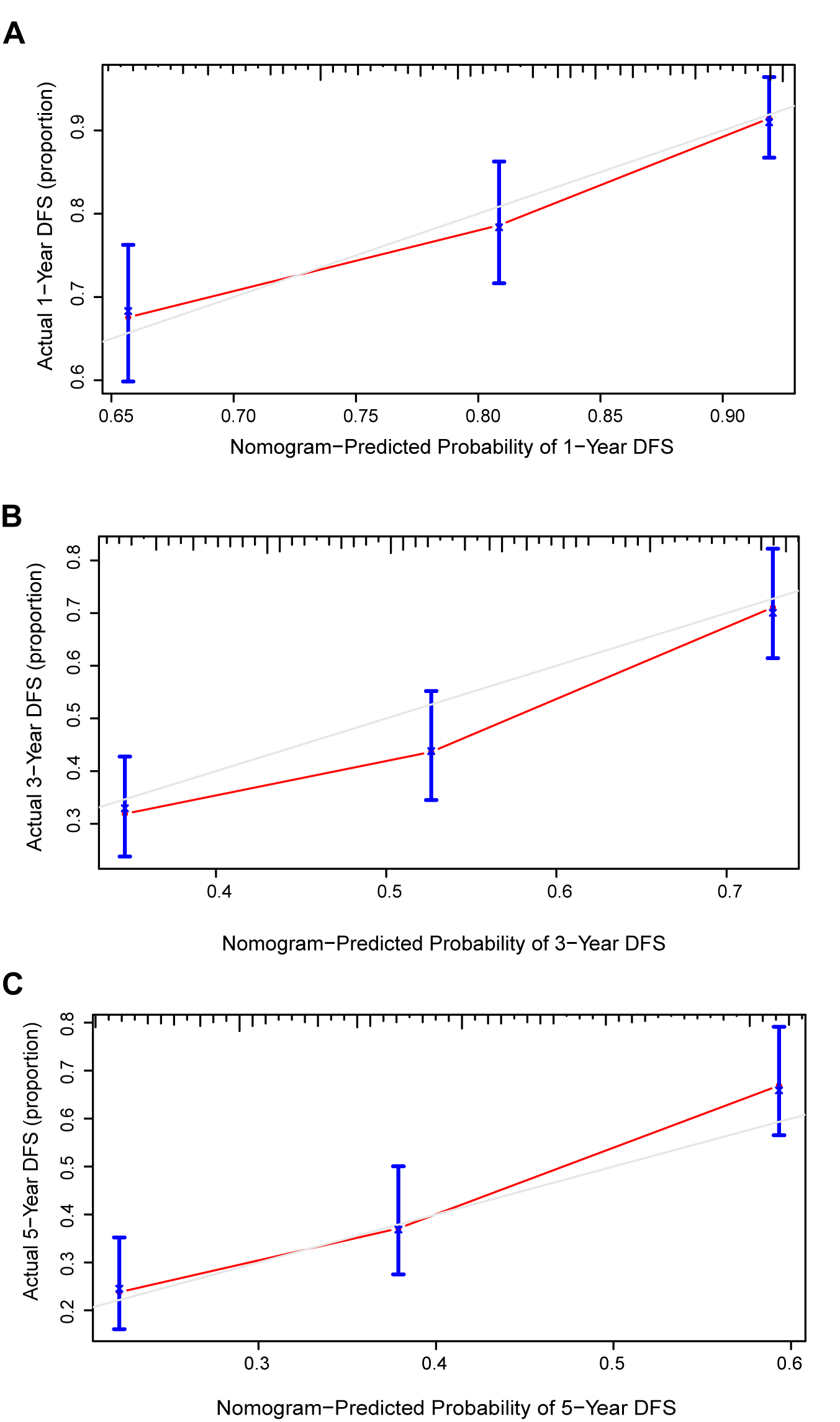
**

Supplement: Supplemental Information 3 [file peerj-13-18911-s003.docx]
