# Supplementary material for: Construction and verification of a prognostic model for bladder cancer based on disulfidptosis-related angiogenesis genes
Source: PeerJ. 2025 Feb 21;13:e18911. doi: 10.7717/peerj.18911 (PMC11849515; doi:10.7717/peerj.18911)
Supplement: Supplemental Information 8 [file peerj-13-18911-s008.docx]

**Table S3: Clinical information of two subgroups**

| **TCGA-BLCA cohort（N=401）** | **K=1(N=203)** | **K=2(N=198）** |
| --- | --- | --- |
| **Age** |  |  |
| <65 | 82 | 67 |
| >=65 | 121 | 131 |
| **Gender** |  |  |
| male | 163 | 134 |
| female | 40 | 64 |
| **Stage** |  |  |
| I | 2 | 0 |
| II | 61 | 63 |
| III | 70 | 71 |
| IV | 70 | 62 |
| **T** |  |  |
| 1 | 2 | 1 |
| 2 | 56 | 59 |
| 3 | 96 | 107 |
| 4 | 33 | 28 |
| **N** |  |  |
| 0 | 113 | 123 |
| 1 | 23 | 23 |
| 2 | 41 | 35 |
| 3 | 4 | 3 |
| **M** |  |  |
| 0 | 100 | 94 |
| 1 | 5 | 5 |
| **Race** |  |  |
| White | 163 | 157 |
| Asian | 21 | 22 |
| Black or african american | 10 | 12 |
| Not reported | 9 | 7 |
